# Endovascular Treatment of Two Pseudoaneurysms Originating From the Left Ventricle

**DOI:** 10.1007/s00270-012-0540-8

**Published:** 2013-01-19

**Authors:** Wojciech Cwikiel, Inger Keussen, Ronny Gustafsson, Arash Mokhtari

**Affiliations:** 1 Department of Radiology, Skane University Hospital, 22185 Lund, Sweden; 2 Department of Thoracic Surgery, Skane University Hospital, Lund, Sweden

## Abstract

A 67-year-old woman resented with an acute type A aortic dissection, which was treated surgically with aortic valve replacement as a composite graft with reimplantation of the coronary arteries. At the end of surgery, a left-ventricular venting catheter was placed through the apex and closed with a buffered suture. Consecutive computed tomography (CT) examinations verified a growing apex pseudoaneurysm. Communication between the ventricle and the pseudoaneurysm was successfully closed with an Amplatz septal plug by the transfemoral route. Follow-up CT showed an additional pseudoaneurysm, which also was successfully closed using the same method.

## Case Report

**Fig. 1:**
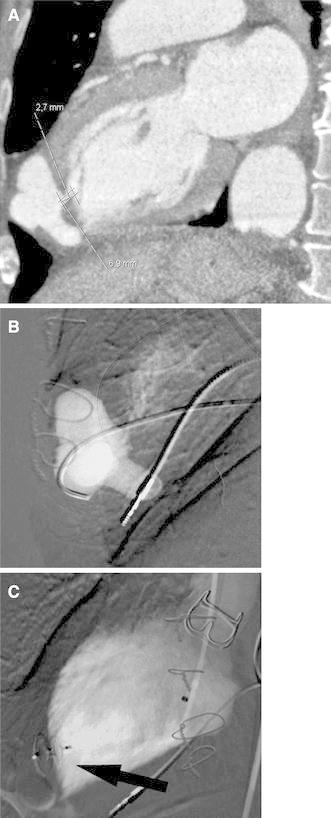
Apical pseudoaneurysm. **A** CT showing apical pseudoaneurysm. **B** Catheter positioned in the pseudoaneurysm at angiography. **C** Exclusion of the pseudoaneurysm by amplatzer septal occluder (*arrow*)

A 67-year-old woman with known hypertension, otherwise healthy, was admitted to our hospital for acute chest pain. A suspicion of myocardial infarction was not confirmed, and computed tomography (CT) showed a type A aortic dissection with a large intramural haematoma in the ascending aorta. Surgical repair was performed urgently with implantation of a biological aortic valve as a composite graft in the ascending aorta with reimplantation of coronary arteries by way of Bentall procedure. The surgery, performed with extracorporeal circulation, hypothermia, circulatory arrest, and retrograde cerebral perfusion, lasted for 21 min. At the end of surgery, the patient showed signs of right heart failure, possibly caused by air embolization to the right coronary artery. Extracorporeal perfusion was reinstituted, and a 14F venting catheter was placed through the apex of the left ventricle. The catheter was subsequently removed and the hole in the apex wall closed with a suture. The postoperative period was uncomplicated with the exception of a pacemaker implantation due to atrioventricular block II. Consecutive CT examinations, performed 1 and again at 2 months after surgery, showed slowly increasing size of the pseudoaneurysm extending from the apex of the left ventricle (Fig. 1A) and a minor septum defect. An echocardiogram confirmed the findings, and additional CT also showed a minor leakage from the aortic graft suture into the false lumen of the dissection. Due to expected technical difficulties with surgical repair in this otherwise stable patient, endovascular closure of the pseudoaneurysm was attempted.

The procedure was performed with the patient under general anesthesia and prepared for emergency surgery in the hybrid angiography/operating room. After puncture of the right common femoral artery using a micropuncture set, a 6F introducer sheath was inserted. A 5F, 100-cm Cobra catheter (Cook Medical, Bjaeverskov, Denmark) was used jointly with a 0.035-inch glidewire (Terumo, Tokyo, Japan) to negotiate through the aortic valve to the left ventricle. Angiography verified open communication to the large, 4.5 × 2.5–cm pseudoaneurysm. However, due to heart movements, visualization of the exact localization of the channel to the pseudoaneurysm at angiography was not possible. After exchange over the wire, a 5F H1 catheter (Cordis, Johnson & Johnson, Miami, FL) was used jointly with the glidewire to navigate into the lumen of the pseudoaneurysm. During relatively time-consuming manipulations, several episodes of ventricular arrhythmia occurred, which, however, were under control due to the function of the pacemaker. After the tip of the catheter was positioned in the pseudoaneurysm, angiography was performed (Fig. 1B). The catheter was exchanged over the 260 cm long, 0.035-inch Amplatz wire (Boston Scientific, Natic, MA) to the 6F inner diameter guiding catheter (Boston Scientific). Through the guiding catheter, a 5-mm Amplatzer septal occluder (AGA Medical, Plymouth, MN) was advanced to the psudoaneurysm. After deployment of the distal part, the occluder and the guiding catheter were jointly pulled back until the operator could feel resistance, and then the other part of the occluder was deployed and the device released (Fig. 1C). Angiography performed after approximately 10 min verified occlusion of the channel to the pseudoaneurysm as well as a persistent small leakage, which was expected to close spontaneously. All of the devices were removed, and the puncture site in the right common femoral artery closed by manual compression.

**Fig. 2:**
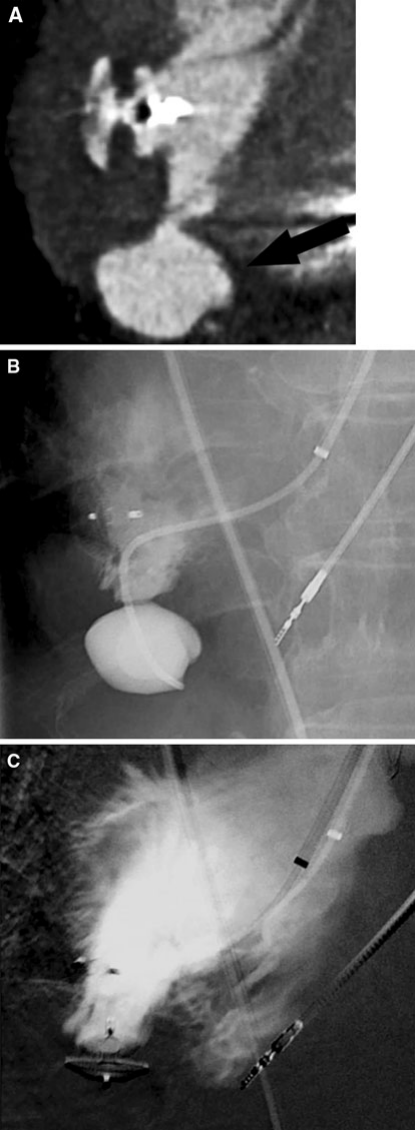
Second pseudoaneurysm at the bottom of the apex. **A** CT after 1 week demonstrates exclusion of the first pseudoaneurysm and the second aneurysm (*arrow*). **B** Angiography catheter tip positioned in the second pseudoaneurysm. **C** The second pseudoaneurysm excluded with the amplatzer

**Fig. 3:**
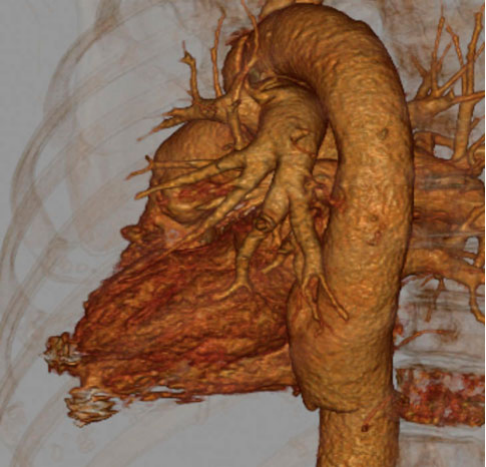
CT performed 3 months after the procedure. Three-dimensional reconstruction confirms exclusion of both pseudoaneurysms

CT performed 10 days later verified absence of contrast flow to the treated pseudoaneurysm; however, the other pseudoaneurysm extending from the base of the apex was visualized (Fig. 2A). This pseudoaneurysm was previously not diagnosed most probably due to compression by the first pseudoaneurysm. A new attempt at endovascular exclusion of the pseudoaneurysm was performed, using the same setting and technique, as described previously (Fig. 2B). Angiography, performed after deployment of the 4-mm Amplatz septal occluder in the second pseudoaneurysm, showed successful exclusion (Fig. 2C). Again, hemostasis was obtained by manual compression of the access site to the femoral artery after removal of all devices. The patient was discharged the day after procedure. At the next CT examinations 3 weeks and 3 months after the procedure, total exclusion of both pseudoaneurysms was seen (Fig. 3), and the patient was at this time in healthy condition.

## Discussion

The mortality rate of patients with type A aortic dissection is high, and emergency surgical repair is mandatory. Symptoms of type A aortic dissection may be similar to those of heart infarction or other serious thoracic disorders. The diagnosis is usually obtained by echocardiogram and CT/magnetic resonance imaging. Surgical repair with extracorporeal circulation in hypothermic circulatory arrest is warranted [1, 2]. Replacement of both the aortic valve and the aortic root may be necessary [3].

Transapical catheterization of the left ventricle is the common access route for catheter placement for venting or unloading of the left ventricle and currently also for replacement of the heart valve [4, 5, 10]. Development of an apical pseudoaneurysm after cannulation is rare, but it has been described in several case reports [6–14]. The pseudoaneurysm is located inside the heart muscle, which makes surgical repair difficult, and a part of the apex must often be resected [10]. Conservative treatment may also be attempted [6], but in cases of progressive enlargement of the pseudoaneurysm, invasive treatment is necessary.

In our patient, the pseudoaneurysm appeared on CT examination 1 month after surgery. At the next CT examination 1 month later, the pseudoaneurysm increased had in size. Surgical repair was considered potentially difficult and unsafe; thus, endovascular treatment was performed. To avoid any possible risk the procedure was performed in the hybrid angiography/operating room [19] with patient prepared for open surgery. The second pseudoaneurysm was not seen at the end of the first procedure. It could have arisen secondary to compression by the primarily treated pseudoaneurysm or it may have developed later due to fragility of the ventricular wall.

Endovascular exclusion of the cardiac pseudoaneurysm has been described [14–18]; however, to our knowledge, closure of two ventricular pseudoaneurysms in the same patient has not been reported.

## Conclusion

We conclude that endovascular treatment of multiple apical ventricular pseudoaneurysms with a septal occluder is feasible and may be performed without complications and with excellent results.
